# Modeling-informed Engineered Genetic Incompatibility strategies to overcome resistance in the invasive *Drosophila suzukii*

**DOI:** 10.3389/finsc.2022.1063789

**Published:** 2022-11-22

**Authors:** Adam Sychla, Nathan R. Feltman, William D. Hutchison, Michael J. Smanski

**Affiliations:** ^1^ Department of Biochemistry, Molecular Biology, and Biophysics, University of Minnesota, Saint Paul, MN, United States; ^2^ Biotechnology Institute, University of Minnesota, Saint Paul, MN, United States; ^3^ Department of Entomology, University of Minnesota, Saint Paul, MN, United States

**Keywords:** genetic biocontrol, agent-based modeling, spotted wing drosophila, resistance, incompatible insect technique (IIT)

## Abstract

Engineered Genetic Incompatibility (EGI) is an engineered extreme underdominance genetic system wherein hybrid animals are not viable, functioning as a synthetic speciation event. There are several strategies in which EGI could be leveraged for genetic biocontrol of pest populations. We used an agent-based model of *Drosophila suzukii* (Spotted Wing Drosophila) to determine how EGI would fare with high rates of endemic genetic resistance alleles. We discovered a surprising failure mode wherein field-generated females convert an incompatible male release program into a population replacement gene drive. Local suppression could still be attained in two seasons by tailoring the release strategy to take advantage of this effect, or alternatively in one season by altering the genetic design of release agents. We show in this work that data from modeling can be utilized to recognize unexpected emergent phenomena and *a priori* inform genetic biocontrol treatment design to increase efficacy.

## Introduction

In genetic biocontrol, the pest organism is genetically manipulated to produce biocontrol agents that act as pesticides. Intentional release of these agents into the environment with numbers and intervals specifically tailored for the technology used and goals of the campaign, suppresses local pest populations in a species-specific manner. Several strategies for genetic biocontrol have been described, and each has unique strengths and weaknesses regarding the release numbers required, the susceptibility to evolved or endemic genetic resistance, and the degree of population control that is attainable (1–10), as reviewed in Alphey et al. (10).

**Figure 1:**
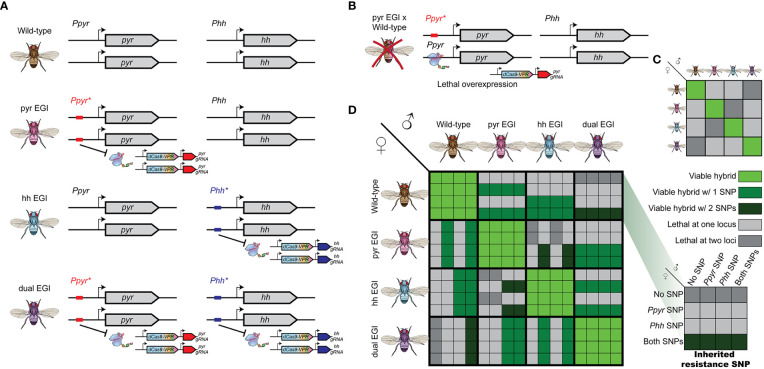
Genetic design and cross-compatibility of Engineered Genetic Incompatibility (EGI) in presence of natural resistant variants **(A)** EGI agents are homozygous for haplosufficient Cas9-activators targeting a developmental regulator, whose ectopic expression is lethal. A haploinsufficient mutation in the promoter target provides resistance. Orthogonal EGI can be generated by targeting different developmental regulators (middle). A single EGI line can have multiple independent targets (bottom). **(B)** Hybrids with wild-type contain both the Cas9-activator and a sensitive allele leading to lethal overexpression. **(C)** Summarized cross compatibility between strains assuming no natural resistance. **(D)** Detailed cross compatibility of between strains when natural resistant alleles exist in the target population. Large boxes indicate parental strains. Inset boxes represent which if any natural resistant SNPs are deposited by the parental strains (inset right).

Engineered Genetic Incompatibility (EGI), a form of extreme underdominance, can form the basis for several types of genetic biocontrol. EGI organisms are homozygous for two genetic elements: a haplosufficient lethal allele and haploinsufficient resistance allele (**Figures 1A, B**) (5, 6, 8, 11). EGI populations are true-breeding with no loss of fecundity. When EGI individuals mate with wild-type individuals, the hybrid offspring receive a copy of the lethal allele and a copy of the resistance allele. Since the resistance allele is haploinsufficient, these hybrids are inviable.

Recent demonstrations of EGI in model organisms use sequence-programmable transcriptional activators (PTAs) to induce lethal over- or ectopic-expression of tightly regulated genes (5, 6, 8). Resistance in the EGI line is provided by small indel mutations that prevent PTA binding to the target promoter.

EGI has practical applications for both population suppression and population replacement. If only male EGI agents are released, the program would mimic other incompatible insect technique (IIT) approaches (12), as reviewed by Alphey et al. and Lees et al. (13, 14). EGI males mate with wild-type females to produce inviable offspring, lowering the overall population in the next generation. Alternatively, if both male and female EGI agents are released, it functions as a threshold dependent gene drive. Assuming equal fitness, when EGI agents outnumber wild-type in a randomly mating population, they are more likely to mate with their own type and have viable offspring (15), reviewed in Sinkins et al. (16). Over time, EGI would grow in proportion until the wild-type population is completely replaced. The opposite occurs if wild-type individuals outnumber the EGI agents. A difference in the relative fitness of EGI and wild type individuals would change the value of the threshold for population replacement (e.g., from 50% to 40%).

For any genetic biocontrol approach, a thorough understanding of the causes and consequences of endemic or evolved genetic resistance is prerequisite to developing field applications. It can lead to genetic design strategies to lower the likelihood of genetic resistance evolving (17, 18). Alternatively, it can lead to release strategies that mitigate the impact of genetic resistance.

While the mechanisms and consequences of resistance to traditional chemical pesticides, herbicides, or antibiotics are different from resistance to genetic biocontrol, population-level strategies to overcome resistance are still informative. Theoretical work has shown that, for two antibiotics that target different essential pathways (i.e., with different mechanisms of resistance), using both antibiotics simultaneously will better combat resistance than using them in an alternating manner (19). Importantly, this literature does not consider negatively-correlated cross resistance. Negatively-correlated cross resistance describes a scenario where genetic resistance to one pesticide increases susceptibility to another, and vice versa. Applying two pesticides with negatively-correlated cross resistance in an alternating fashion would prevent the selection for resistant genotypes and allow for long-term stability for pest management efforts. Unfortunately, negatively-correlated cross resistance is rare among resistance to clinically used antibiotics and chemical pesticides (20), as reviewed in *David* (21). However, unlike chemical pesticides, genetic biocontrol agents can transfer new genetic material into the populations they are targeting. This offers new opportunities for rationally engineering negatively-correlated cross resistance.

We recently demonstrated the rational engineering of multiple mutually-incompatible EGI fruit flies. Each EGI genotype produced 100% inviable offspring when crossed to wild-type or to other EGI genotypes (6). We hypothesized (and explore in this study) that alternating release of two mutually incompatible EGI agents could generate negatively-correlated cross resistance and lead to stable population suppression in the face of genetic resistance.

Each EGI strain (for example one targeting the developmental morphogen *pyramus* (pyr) and another targeting the developmental morphogen *hedgehog* (hh)) would carry alleles susceptible to targeting from the other (**Figure 1A**). If endemic resistance alleles in the wild population allow survival of hybrid offspring between *pyr* EGI and wild-type parents, those offspring necessarily inherit at least one susceptible allele to the *hh* EGI agent. We compare this to a single EGI strain with two independent, individually lethal targets (dual EGI), an analogue to combination therapy applied to microbes (**Figure 1A** bottom). In order to escape lethality, a hybrid offspring must carry two independent resistance alleles.

In this manuscript, we use an agent-based model for invasive seasonal Spotted Wing Drosophila (*Drosophila suzukii*, SWD) populations to assess the sustainability of an EGI-based control strategy. This fruit fly spread quite rapidly from Asia to the Americas and Europe within a brief 5 years, and has caused major economic losses as a pest of multiple berry crops, reviewed in Asplen et al. (22). Although multifaceted pest management programs have been developed in some countries, little attention has focused on genetic biocontrol strategies for SWD, as reviewed in Tait et al. and Venette & Hutchison (23, 24).

We use our modeling to predict how the design and release strategy of EGI-based biocontrol will fare when challenged with high rates of endemic genetic resistance. Since only female SWD are damaging, we apply EGI in a manner similar to the ITT approach, with only males released to suppress local populations (9). We compare EGI designs with a single-gene target and with dual-gene targets, simulated in several release strategies. We report a strategy of population replacement followed by suppression that is robust to resistance through natural variation. Furthermore, we used the emergent results to inform a new round of genetic design work that overcomes the primary modes of genetic resistance.

## Methods

### Agent-based modeling

In this study, we used an agent-based model of SWD that we previously described, publicly available *via* GitHub (https://github.com/smanskiLab/SWD_Agent_Model) (9). Broadly, this agent-based model tracks individual genotypes at an arbitrary number of genetic loci. For the simulations in this work we used seven diploid loci (**Table 1**). We generated natural resistance at loci 0 and 2 in the wild population with a frequency of Freq1 (0.01) and Freq2 (0.01) respectively. The beginning of the season sees a population decline that is important for admixing the agent lifestages. To prevent a genetic bottleneck from artificially removing or concentrating the resistance alleles, they are generated at timestep, genSNP, 32 in our simulation.

**Table 1:** Description of the possible alphabetical values and their meaning for each position in the 7-digit alphabetical code representing possible genotypes in the agent-based model.

Locus	Value: meaning	Value: meaning	Value: meaning
0	b: wild-type	B: engineered promoter mutant	c: natural resistant SNP
1	d: wild-type	D: PTA targeting locus 0	
2	p: wild-type	P: engineered promoter mutant	r: natural resistant SNP
3	t: wild-type	T: PTA targeting locus 2	
4	l: wild-type	L: female lethal linked to locus 1	
5	W: wild-type	F: female lethal linked to locus 3	
6	X: recessive female phenotype	Y: dominant male phenotype	

Key genotypes (alleles in **Table 1**) of this experiment are: wild-type, bbddppttX(X/Y); pyr EGI Strain, BBDDppttllWWX(X/Y); hh EGI Strain, bbddPPTTllWWX(X/Y); dual EGI Strain, BBDDPPTTllWWX(X/Y); pyr L-SSIMS Strain, BBDDppttLLWWX(X/Y); hh L-SSIMS Strain, bbddPPTTllFFX(X/Y); dual L-SSIMS Strain, BBDDPPTTLLFFX(X/Y).

In the last set of L-SSIMS simulations, we had the D, T, L, and F alleles independently revert to d, t, l, and W respectively in 10% of eggs that inherited the allele.

We further modified the code to enable alternating between which strains were released. The command line was used to direct the remaining parameters in the form: python [code file].py [timesteps between releases] [males/release] [directory for results files] [starting EGI genotype] [releases between alternating strains]. We made the source code used in this publication publicly available *via* GitHub (https://github.com/smanskiLab/SWD_Resistance_Model) to allow for reproducibility of other groups who would like to make use of this model.

For each condition we ran ten replicate simulations on 1TB RAM nodes of the Mesabi Cluster at the Minnesota Supercomputing Institute.

### Data analysis

Cumulative and timestep specific data points were reported as the arithmetic mean of ten replicate simulations and all error bars represent one standard deviation from the mean.

An ANOVA was used to identify significance between suppression values. For those with significance, p-values were calculated with the Student’s t-test. Plots on a logarithmic scale included a pseudo-count of one (i.e. 0 is depicted as 1, 10 is depicted as11, etc.).

To generate the logistic curves, we used the Levenberg–Marquardt algorithm to fit the function


$$y=1/\left(1+e^{-k*\left(x-x_{0}\right)}\right)$$

, where x is the timestep, k is the steepness constant, x _0_ is the midpoint for the logistic.

## Results

### Combined EGI systems are more robust to resistance caused by natural sequence diversity

It is difficult to test the impact and mitigation of genetic biocontrol resistance mechanisms in a lab setting where space, time, and resolution of measurement is limited. We previously described an agent-based model of SWD for comparison of biocontrol techniques in the field (9) and apply it here to investigate resistance propagation and mitigation under a range of EGI release strategies. This model tracks each SWD agent individually, complete with genotype and stochastic, temperature-dependent development progression. Seasonal temperature data from St. Paul, MN and published information on SWD development and fecundity serve as the basis for the probabilities used while offspring are generated using Mendelian inheritence from their parents.

Simulated populations with control strategy follow highly reproducible seasonal dynamics. From a low point in early April, the population grows to the order of 10^4^ in early July. Subsequently, the population drops due to high temperatures (**Supplementary Note 2** bottom). The simulation does not account for food availability or population density but matches expected annual trends. We assume an isolated population to focus on population dynamics resulting from resistance within a targeted release area. Based on the maximum population, we estimate that it is simulation of a 4 ha field (25).

**Figure 2:**
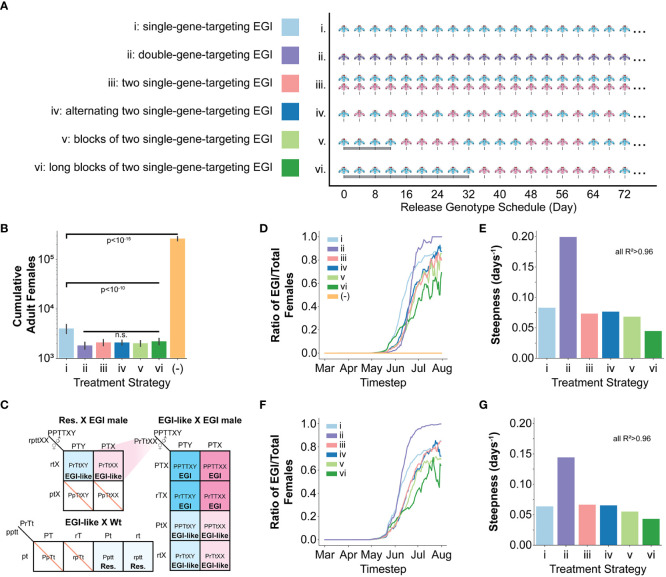
Modeling one season of SWD in the presence of EGI based IIT and natural sequence diversity generated resistance. **(A)** Six male only EGI release strategies were tested using the pyr EGI (blue), hh EGI (red), and dual EGI strains (purple). **(B)** Total counts of adult female SWD agents over the course of the simulation of one season with initial seeding of resistant alleles, each independently generated at 1% frequency. Resistant alleles were seeded on April 1st. **(C)** Expanded Punnett square showing genetic outcomes of mating between a resistant agent and an EGI male. The mating allows for generation of EGI females that can then drive a population replacement. P=Engineered resistant promoter, p=wild-type promoter, r=resistant SNP, T=PTA, t=wild-type sequence X=recessive female phenotype, Y=dominant male phenotype) **(D, E)** Data collected from simulation of one season with initial seeding of resistant alleles, each independently generated at 1% frequency. **(F, G)** Data collected from simulation of one season with initial seeding of double homozygous resistant agents at 1% frequency. **(D, F)** Ratiometric tracking of EGI relative to the total number of agents at each timestep throughout the season. **(E, G)** Steepness parameter from logistic curve fit to data in **(D, F)**. (See also **Supplementary Note 4**.). ANOVA was used to identify suppression values that significantly differ from each other. A Student's t-test was used to calculate p-values for those that did.

We simulated six different male only release IIT genetic biocontrol scenarios (**Figure 2A**): (i) iterative release of a single-gene-targeting EGI agent (pyr EGI), (ii) iterative release of a double-gene-targeting EGI agent (where only one target must be present in the wild mate for complete hybrid lethality) (dual EGI), (iii) iterative release of a mixed population of two unique single-gene-targeting EGI agents (pyr EGI and hh EGI), and (iv, v, vi) iterative and alternating release of two single-gene-targeting EGI agents (pyr EGI and hh EGI) with rapid, medium, or long-period cycling of the two genotypes. We chose pyr and hh as named targets for ease of tracking in the paper but do not model any unique characteristics for these targets, except that they provide 100% hybrid lethality in a susceptible genotype. These two EGI designs function identically in our model and other targets could be used for implementation in live organisms.

In each case, we used a high rate of natural genetic resistance (1%) to ensure that resistance emerges in simulations with moderate population sizes. With an allele frequency of 1% and two independent diploid target loci, roughly 4% of the population contains at least one resistant allele. This frequency of resistance is much higher than the rate of *de novo* evolved resistance, which has not been observed in the laboratory after assessing thousands of offspring (6, 9). However, it is possible for this rate of endemic resistance to exist if PTA binding sites are not selected strategically (26).

Release of a single target EGI had 85% reduction on number of females over the course of a growing season. Notably, all of the release strategies that include two targets, separately or in a single agent, exhibited an additional suppression effect but did not significantly differ from each other (**Figure 2B**).

In examining the impact of resistance alleles, it is useful to separately consider resistance alleles in a wild-type background from those in the background of a PTA-expressing strain. When mating with a single-targeting EGI male, females harboring at least one copy of the resistant allele would lay eggs with “EGI-like” genotypes, that are resistant to EGI but are not homozygous for the PTA (**Figure 2C**). If any EGI-like females mate with an EGI male, 25% of the offspring would be fully EGI females. EGI males, EGI-like males, and EGI-like females would each represent an additional quarter of the offspring, and these are all viable genotypes. EGI-like matings with wild-type regenerate the resistant genotype. Similar regeneration can occur in the dual targeting EGI but requires at least one copy of both resistant alleles to be present in a single female agent.

### Resistance leads to a threshold dependent gene drive phenomenon

We found that the resistant allele frequency in a wild-type genetic background remains relatively stable for the first 10 weeks and then drops to 0 by approximately early July (**Supplementary Note 1A**). The dual-targeting EGI agent drove moderately lower levels of resistance allele in wild-type backgrounds during this time, but still reached 0 at the same time. Interestingly, the single-target EGI strain used in isolation saw a decrease in resistance frequency in the wild-type genetic background approximately 4 weeks sooner than the other approaches. However, closer examination shows that this is due to the fate of the resistance allele in EGI populations.

The largest impact of genetic resistance in each population control strategy is the creation of population replacement gene-drive behavior through in-field creation of females with EGI genotypes (despite no EGI females being released) (**Figures 2C, D**; **Supplementary Note 1B**, **2**). These further suppressed wild populations but allowed the EGI strains to propagate. In essence, the intended IIT strategy becomes a population replacement drive. The dual-targeting EGI genotype showed an interesting behavior. Initially, it delayed the emergence of EGI females compared to the other techniques (**Figure 2D**, June). However, the population replacement reliably occurred more rapidly with this genotype (**Figure 2D**, July). We quantified this observation by fitting each population frequency curve from **Figure 2D** to a logistic function and plotting the steepness in **Figure 2E**. All fits gave R^2^ values >0.96 (**Supplementary Note 3A**).

For these fits, we allowed for two free parameters, k (days^-1^, steepness) representing the rate of the driving behavior and x _0_ (days, midpoint) when half of female agents are EGI. The dual-targeting strain had a far steeper growth curve than the other methods (**Figure 2E**; **Supplementary Note 4A**). Even though EGI females are generated later in the season for the dual-targeting strain, the greater growth rate leads to the x _0_ being earlier than any of the mixed release strategies (**Supplementary Note 3A**).

Our simulations generated the resistant alleles stochastically and independently, meaning that double resistance in the wild-type background is unlikely given starting population numbers in our simulations. We reasoned that the efficacy of dual-targeting EGI treatment was dependent on the rarity of a double resistant SWD mating with the male EGI. However, once the cross does occur, there is no mechanism to inhibit spread. In contrast, the alternating release strategies are more likely to generate EGI females in the first place but are efficacious because they continue to inhibit each others’ spread. We hypothesized that this would make the strategies employing two mutually-incompatible EGI strains more robust to presence of double resistance.

To test this, we repeated the simulations but seeded individuals homozygous for the both resistant alleles at a 1% frequency. In this case, EGI females arose for the dual-targeting strategy at a time comparable to a single-targeting release (**Figure 2F**; **Supplementary Note 4**). Fitting again to a logistic curve with R^2^ values > 0.96, the dual targeting EGI reached the midpoint 12-21 days sooner than the mixed release strategies (**Supplementary Note 3B**). Of note, the dual-targeting EGI population rapidly grew to fixation, faster than even the single-targeting EGI (**Figure 2G**; **Supplementary Notes 3B**, **4**).

### Threshold dependent gene drive effect establishes a homozygous population more susceptible to genetic biocontrol

As the replacement gene drive behavior leads to a larger portion of flies being EGI, the fraction of unproductive matings goes down, inhibiting the net efficacy of the suppression. A female population rebound towards the end of the season can be seen in each treatment group in early June (**Supplementary Note 2**, **4**). Furthermore, if overwintering contributes to the next season’s population genetics (as growing evidence suggests), the effect would interact with future treatments (26, 27). To model a second season, we ran the simulation as before but seeded 5, 50, or 95% of either pyr EGI Strain or dual EGI Strain (0.5 sex ratio), representing low, medium, and high rates of overwintering after treatment and population replacement. We then released EGI males with the six previous treatment strategies. We further added strategies akin to i, iv, v, and vi but switched the order of release for pyr EGI Strain and hh EGI Strain (denoted i_r, iv_r, v_r, vi_r) so that the first release in season two was incompatible with the overwintering genotype (**Supplementary Note 5**).

**Figure 3:**
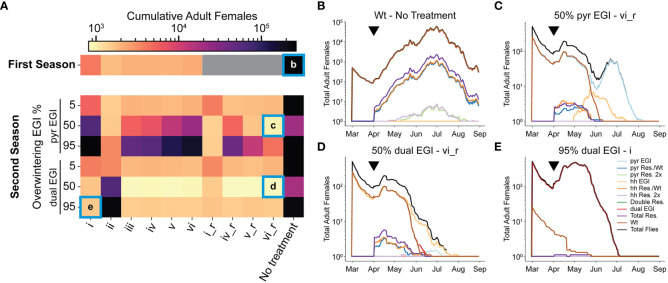
Second season of EGI IIT treatment assuming overwintering. A second season was simulated with 5, 50, or 95% of the starting population being either pyr EGI or dual EGI. **(A)** A log scale heatmap with cumulative counts of adult females in the simulation averaged from 10 replicates. Grey boxes indicate simulations that were not run. **(B–E)** A mapping of genotypes from selected conditions over the course of the full season. Black triangles indicate the timepoint that resistant alleles were generated. New EGI was released five timesteps later. “_r” stands for “reverse”, and denotes strategies where pyr and hh EGI are switched for all releases (i.e. when pyr EGI is release in strategy iv, hh EGI is release in strategy iv_r and vise versa).

With a low (5%) initial frequency of either EGI strain, the treatment mostly reflected that of the first season. The underdominance gene drive behavior of the engineered strains works against the EGI flies that overwintered and they are replaced by wild-type early in the season. This has minimal impact on the overall population size (**Figure 3A**).

When EGI flies represent a moderate or high initial frequency in the population (50% or 95%), the performance of different treatment strategies is more nuanced. As expected, there is little population suppression when the second year’s treatment includes the overwintering strain (e.g. **Figure 3A**, i, ii). Since compatible females already exist in the simulated area, population replacement occurs early. Mixed releases do exhibit better suppression than single strain releases but still worse than in season one (**Figure 3A**, iii-vi, iv_r-vi_r). However, releases of mutually incompatible strains in season two perform better than the best observed suppression in season one (**Figure 3A**). This is especially true when the dual EGI strain dominates at the beginning of season two and hence most agents carry the both the lethal construct and the resistance. Releasing lines with known susceptibility to the dual EGI strain increases the likelihood that a given mating is unproductive. For example, pyr EGI, and hh EGI suppress wild-type in addition to the cross-suppression between these two and the overwintered dual EGI strain (**Figures 1C, D**). Any remaining resistant insects produce first EGI-like and then EGI offspring, which can then be cross-suppressed by the other present EGI strains.

When EGI constitutes 50% of the starting population, a stronger suppression effect is present compared to when EGI constitutes 95% of the seed population (**Figure 3A**). At 50% the wild-type and EGI begin to cross-suppress immediately (**Supplementary Note 5**). This early suppression maintains low levels of adult females throughout the season (**Figures 3B–D**). New EGI agents are not released until April so the suppressive effect from new releases is delayed.

Importantly, population replacement in the first season (i.e. 95% EGI) enables local eradication in the second season (**Figure 3E**). In the second season of release, female agent eradication could occur as early as July (depending on release strategy) (**Figures 3C**, **D**, and **Supplementary Note 5**). The population replacement the previous season ensures that nearly all SWD in the treatment area are genetically susceptible to the treatment in the second season.

### Genetically linking a female lethal construct to the EGI PTA effectively overcomes mechanisms of resistance

**Figure 4:**
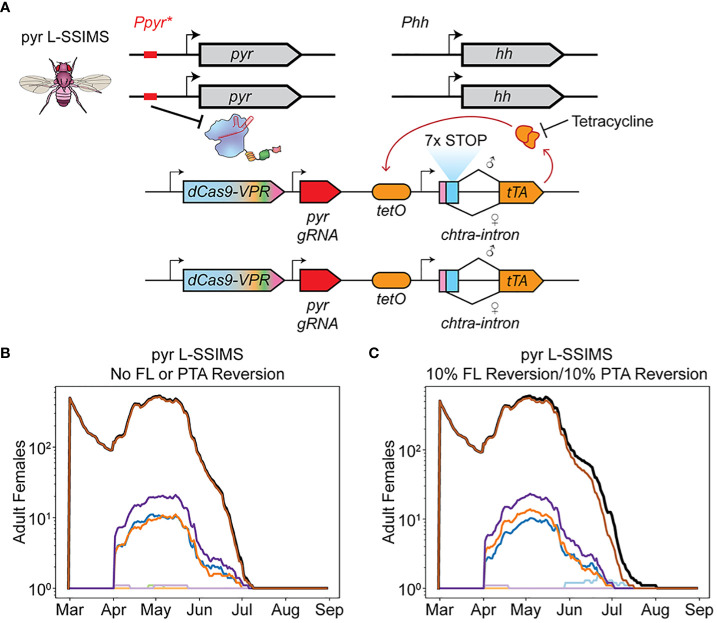
L-SSIMS overcomes resistance mechanisms. **(A)** L-SSIMS genetic design. Sex specific splicing leads to expression of rTA only in females. In absence of tetracycline rTA binds tetO and through positive feedback causes lethal overexpression of rTA. **(B)** Adult females over one season of treatment with male pyr L-SSIMS agents. The FL construct and PTA are never lost. **(C)** Adult females over one season of treatment with male pyr L-SSIMS agents with FL and PTA resistance. The FL construct and PTA are independently reverted to wildtype in 10% of eggs.

Production of female EGI insects in the field drives the population replacement that ultimately inhibits the efficacy of EGI-based population supression. We hypothesized that adding a construct to remove this escaped population to the EGI system would improve the treatment strategy. We previously described a genetic biocontrol technique that combined conditional female lethality (FL) with EGI, sex-sorting incompatible male system (SSIMS) (**Figure 4A**) (7, 9). While the original system has the FL independently assort from the EGI PTA, we realized that if the two were genetically linked it would be a mechanism to clear escapee female EGI (Linked-SSIMS [L-SSIMS]). EGI-like offspring would now carry one copy of the PTA construct linked to one copy of the dominant FL construct and thus only male EGI-like flies would be viable, maintaining the EGI-based IIT.

To confirm the efficacy of such a system, we repeated the treatment i-vi but with a FL construct linked to each PTA (i.e. inheritence of the PTA guaranteed inheritence of the FL).

Regardless of EGI treatment used, the entire female population was eradicated in early July along with the rest of the non-EGI male population (**Figure 4B**, **Supplementary Note 6**).

We simulated the robustness of L-SSIMS to genetic silencing or breakdown of either component. While generating each new egg in our simulation, FL and PTA alleles independently reverted to wild-type 10% of the time. Despite these high failure rates, we saw eradication comparable to running the simulation while ignoring such failure modes (**Figure 4C**). Small numbers of female EGI escapees can and do arise, but they are quickly suppressed by the functional FL alleles that are consistently reintroduced with each release of biocontrol agents.

## Discussion

Experimental and computational research demonstrates the importance of accounting for resistance to genetic biocontrol. For example, replacement, sex-biasing, and suppression threshold independent gene drives exhibit rapid generation and propagation of resistant alleles (18, 28, 29). Meanwhile, dominant female or bi-sex lethal constructs also have reduced performance in a range of simulated and real resistance scenarios (30–32). These data exhibit the need to understand the emergent phenomena associated with genetic biocontrol prior to treatment.

In this work, we used agent based modeling of SWD to investigate the impacts of resistance on EGI-based IIT. We tested a range of genetic design and release strategies to determine pathways for mitigation. We found that natural sequence diversity in the target population may generate EGI females leading to population replacement. Importantly, we found that a second season of EGI based IIT can take advantage of the population replacement effect to provide robust suppression and potentially local eradication. Furthermore, we found that linking a FL construct to the EGI PTA (L-SSIMS) can robustly overcome the main biocontrol resistance mechanisms. The precise population trends of our modeling would not be applicable to other organisms, but we expect that some of the larger scale emergent phenomena and strategies to overcome resistance could be generalized.

Akin to combination therapy applied to combat the emergence of antibiotic resistance, EGI could be generated to target two redundant, independent loci. With full penetrance, a resistant promoter in either target would not be sufficient to prevent hybrid lethality driven by the other target. Our initial report of EGI in *D. melanogaster* uses two guides targeting single loci and demonstrated up to 100% lethality at multiple independent gene targets (5). Combining such designs would readily generate dual-targeting EGI strains. Alternatively, we reasoned that release of two orthogonal EGI strains would function to create negatively-correlated cross resistance. While each EGI strain is individually more susceptible to resistance, any resistant offspring would necessarily carry alleles sensitive to the orthogonal strain (i.e. those inherited from the EGI parent). This second design has the benefit of being easier to generate.

The 1% rate of double resistance modeled is unlikely to occur in nature in absence of selective pressure. In a recent analysis of >10,000 wild SWD, targetable regions can be found with SNP frequencies <0.1% for single targets and could be expected at ≤ 0.2% for 2 targets (26). However, as evidence grows for SWD overwintering, the population replacement behavior of EGI in the late season may allow the treatment of a field one year to seed such genotypes in the next (33, 34). In warmer climates, with year round SWD populations, enduring resistance would be even more damaging to the efficacy of EGI.

With a two season management plan, the population replacement gene drive still allows for localized, targeted eradication. During the first season, a dual-targeting strain can be released. Locally, it would suppress the population. In the early season, there is a suppressive effect of release but in the late season the behavior shifts to population replacement. This latter action is threshold dependent and therefore defines the genotype within a localized region around the release area but cannot spread broadly. Consistent release of EGI males ensures that locally EGI outnumber wild-type, however, the EGI:wild-type ratio would drop as a function of distance from the release site. Once wild-type outnumber EGI the population replacement effect would work toward removing EGI agents.

In the second season, single targeting EGI can be released. These would be non-viable with either the overwintered EGI strain or wild-type individuals leading to strong suppression. Crosses between the new EGI and wild-type SWD, would still be susceptible to the overwintered EGI strain, avoiding propagation of resistance and eventually leading to local eradication. A benefit of this technique is that it is powerful within a local target region while the threshold dependence ensures that non-target areas are largely unaffected. Future modeling work, beyond the scope of this paper, could include migration as a parameter and inform the fine tuning necessary for precise application of this strategy. Such data would further inform strategies for maintaining low levels of SWD populations post suppression.

Our model in this study focuses on natural sequence diversity in the target region as a source of EGI resistance and does not look into spontaneous generation of resistant alleles. Estimates of spontaneous mutation in *D. melanogaster* range from 2.8-7.7 * 10^-9^ per site per generation (35–38). Treating all 40 targeted base pairs plus 4 PAM base pairs independently and as neutral sites, we still expect spontaneous resistance to arise at once in every 2.95 * 10^6^ agents. Impact from natural variance, ranging on the order of 10^-3^-10^-2^ SNPs per base pair, would likely dominate (26). Nonetheless, spontaneous mutations and natural variance confer resistance in a mechanistically identical manner and so we expect the results from this work would be generalizable to practical application where spontaneous mutation is likely to eventually arise.

For most of this work we ignored resistance through silencing or mutation of the haplosufficient lethal PTA because our previous work suggests that this is a minority pathway for novel resistance and there are straight-forward engineering considerations (such as positive selection modules) that would mitigate this pathway (5). Nonetheless, these rarer alternative pathways may lead to unexpected emergent phenomena. For the L-SSIMS simulations, we did examine the most likely modes of resistance: promoter variance, PTA/gRNA silencing/mutation, and FL silencing/mutation.

In large populations, longer-scale ecological evolution may contribute to alternative resistance routes that would not be captured by our simulations. For example, evolution of an assortative mating phenotype would prevent wild insects from mating with the control agents or strain specific penetrance may lead to selection of low-penetrance genotypes that yield more surviving hybrid offspring (31, 39, 40).

Our modeling indicates that even a small number of female EGI are able to start a population replacement drive. This means that segregation between male and female EGI agents prior to release is vital to the efficacy of EGI as an IIT technique, as any accidentally released females could trigger population replacement behavior. SWD display sexual dimorphism that enable manual segregation, though this is not a practical approach at scale. Automated and highly efficient automated sorting has been developed for mosquitoes and the techniques could be potentially adopted for SWD (41). Alternatively, L-SSIMS would enable genetically encoded, automatic sex sorting.

L-SSIMS requires a larger assembly of genetic parts to be brought together and may be a barrier to initial engineering of the strain. Other groups have measured that conditional lethality such as the FL described here have a *de novo* failure rate of 2*10^-5^ (32). FL penetrance is generally strong in different strains of *D. melanogaster*, but has been measured as low as 11% in certain strains (31). Our data demonstrates that L-SSIMS is an effective design for overcoming even higher than expected rates of resistance (up to 10^-1^). Assuming 100% penetrance, L-SSIMS enables rapid eradication of damaging SWD populations in the treatment area. Escapee EGI females are likely to mate with either wild-type (which is unproductive *via* EGI) or L-SSIMS (which would produce more L-SSIMS males but not viable females). After local eradication, L-SSIMS males could be consistently applied as a non-damaging, highly specific preventative treatment.

Previous IIT field applications in other insects have set precedent for large numbers of insects per release, as reviewed in Scott et al. and O’Connor et al. (42, 43). A field trial of radiation sterilized SWD IIT, released 9,000 to 60,000 weekly in 7.5 ha field, a size comparable to our simulated field (25). In comparison, we take a relatively timid EGI release schedule of 800 adults released every four days and maintain this schedule throughout the season (totalling 30,400 released SWD). Lowering release numbers further could suppress populations while ensuring that the escaping EGI are always below the population replacement threshold and may serve to inhibit expansion of resistance.

Through our modeling in this paper, we examine how resistant alleles impact EGI-based IIT and strategies that mitigate such effects. We discover negatively-correlated cross resistance as a viable technique in the context of EGI. Importantly, we observe that resistance leads to a delayed population replacement effect. We find that this can be leveraged if overwintering significantly contributes to the genetics of a second season. Finally, our modeling informed a new round of genetic design to generate L-SSIMS, a biocontrol method that overcomes the primary modes of resistance evolution.

## Data availability statement

The datasets presented in this study can be found in online repositories. The names of the repository/repositories and accession number(s) can be found below: https://github.com/smanskiLab.

## Author contributions

MJS and AS conceptualized the study. MJS and WDH acquired funding. AS and NRF developed the model. AS performed experiments. AS and MJS analyzed data. AS and MJS wrote the manuscript. All authors contributed to the article and approved the submitted version.

## Funding

Funding for this project, including support to AS and NF, was provided by the Minnesota Invasive Terrestrial Plants and Pests Center, through the Environment and Natural Resources Trust Fund, as recommended by the Legislative-Citizen Commission on Minnesota Resources (LCCMR). AS and MS were partially supported by the USDA grant 2021-33522-35340.

## Acknowledgments

We acknowledge the Minnesota Supercomputing Institute (MSI) at the University of Minnesota for providing resources that contributed to the results reported within this paper.

## Conflict of interest

MJS is a cofounder of Novoclade, Inc., and he and NRF holds patents related to the EGI technology.

The remaining authors declare that the research was conducted in the absence of any commercial or financial relationships that could be construed as a potential conflict of interest.

## Publisher’s note

All claims expressed in this article are solely those of the authors and do not necessarily represent those of their affiliated organizations, or those of the publisher, the editors and the reviewers. Any product that may be evaluated in this article, or claim that may be made by its manufacturer, is not guaranteed or endorsed by the publisher.
