# Corrigendum: Modeling-informed Engineered Genetic Incompatibility strategies to overcome resistance in the invasive *Drosophila suzukii*

**DOI:** 10.3389/finsc.2023.1360167

**Published:** 2024-01-12

**Authors:** Adam Sychla, Nathan R. Feltman, William D. Hutchison, Michael J. Smanski

**Affiliations:** ^1^ Department of Biochemistry, Molecular Biology, and Biophysics, University of Minnesota, Saint Paul, MN, United States; ^2^ Biotechnology Institute, University of Minnesota, Saint Paul, MN, United States; ^3^ Department of Entomology, University of Minnesota, Saint Paul, MN, United States

**Keywords:** genetic biocontrol, agent-based modeling, spotted wing drosophila, resistance, incompatible insect technique (IIT)

**Error in Figure/Table**

In the published article, there was an error in **Table 1** as published. The Value: meaning at Locus 2 (i.e., second column from left, third row below header) was erroneously listed as “r: wild-type”. Instead, it should have been listed as “p: wild-type“. The corrected **Table 1** and its caption appear below.

**Table 1:** Description of the possible alphabetical values and their meaning for each position in the 7-digit alphabetical code representing possible genotypes in the agent-based model.

Locus	Value: meaning	Value: meaning		Value: meaning
0	b: wild-type	B: engineered promoter mutant		c: natural resistant SNP
1	d: wild-type	D: PTA targeting locus 0		
2	p: wild-type	P: engineered promoter mutant		r: natural resistant SNP
3	t: wild – type	T: PTA targeting locus 2		
4	1: wild-type	L: female lethal linked to locus 1		
5	W: wild-type	F: female lethal linked to locus 3		
6	X: recessive female phenotype	Y: dominant male phenotype		

The authors apologize for this error and state that this does not change the scientific conclusions of the article in any way. The original article has been updated.

