# Supplementary material for: Modeling-informed Engineered Genetic Incompatibility strategies to overcome resistance in the invasive Drosophila suzukii
Source: Front Insect Sci. 2022 Nov 22;2:1063789. doi: 10.3389/finsc.2022.1063789 (PMC10926386; doi:10.3389/finsc.2022.1063789)
Supplement: Supplementary file 1 [file Presentation_1.pdf]

Supplementary Note 1: Resistant allele frequencies in populations lacking the PTA

Supp Note 1. a) Persistence and stability of resistant alleles within the PTA-free population. This was calculated by the number of resistant alleles present among both loci when a PTA was not present divided by the total number of agents at that timestep. Untreated simulations maintained an approximately constant rate of resistant alleles as expected. Simulations that included EGI release maintained comparable but distinct patterns early in the season. However, the resistant population eventually crashed between June and July. b) The average count of each genotype among adult females was tracked at each timestep under one season of treatment with 1% presence of resistant SNPs. The graphs depict each genotype normalized to the total number of adult female agents in that timestep.

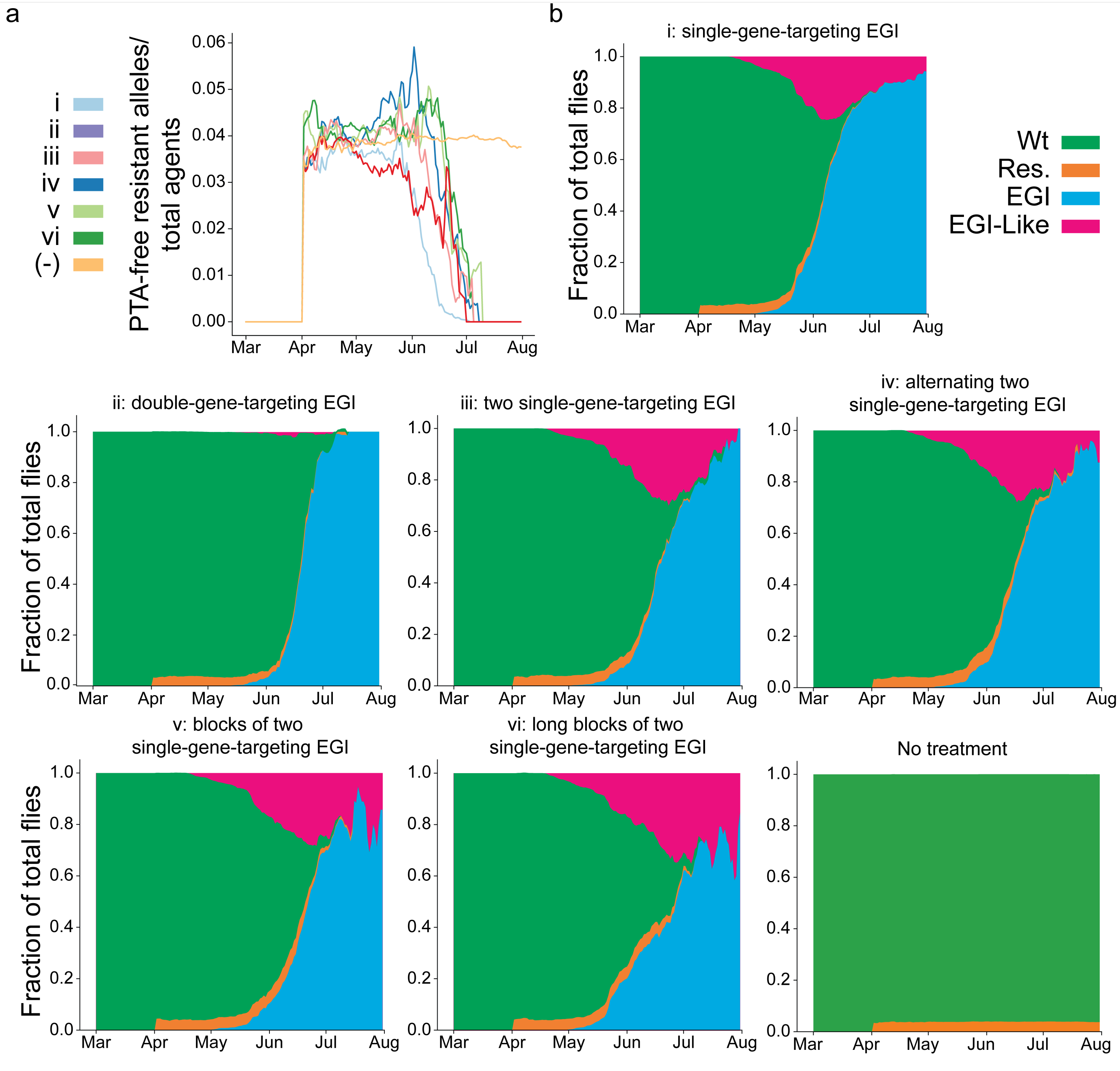

Fig. 5

Supplementary Note 2: Traces of adult genotypes over one season of treatment

Supp Note 2. Average counts of genotypes over one season seeded with 1% resistant allele frequency and treated according to Figure 2a.

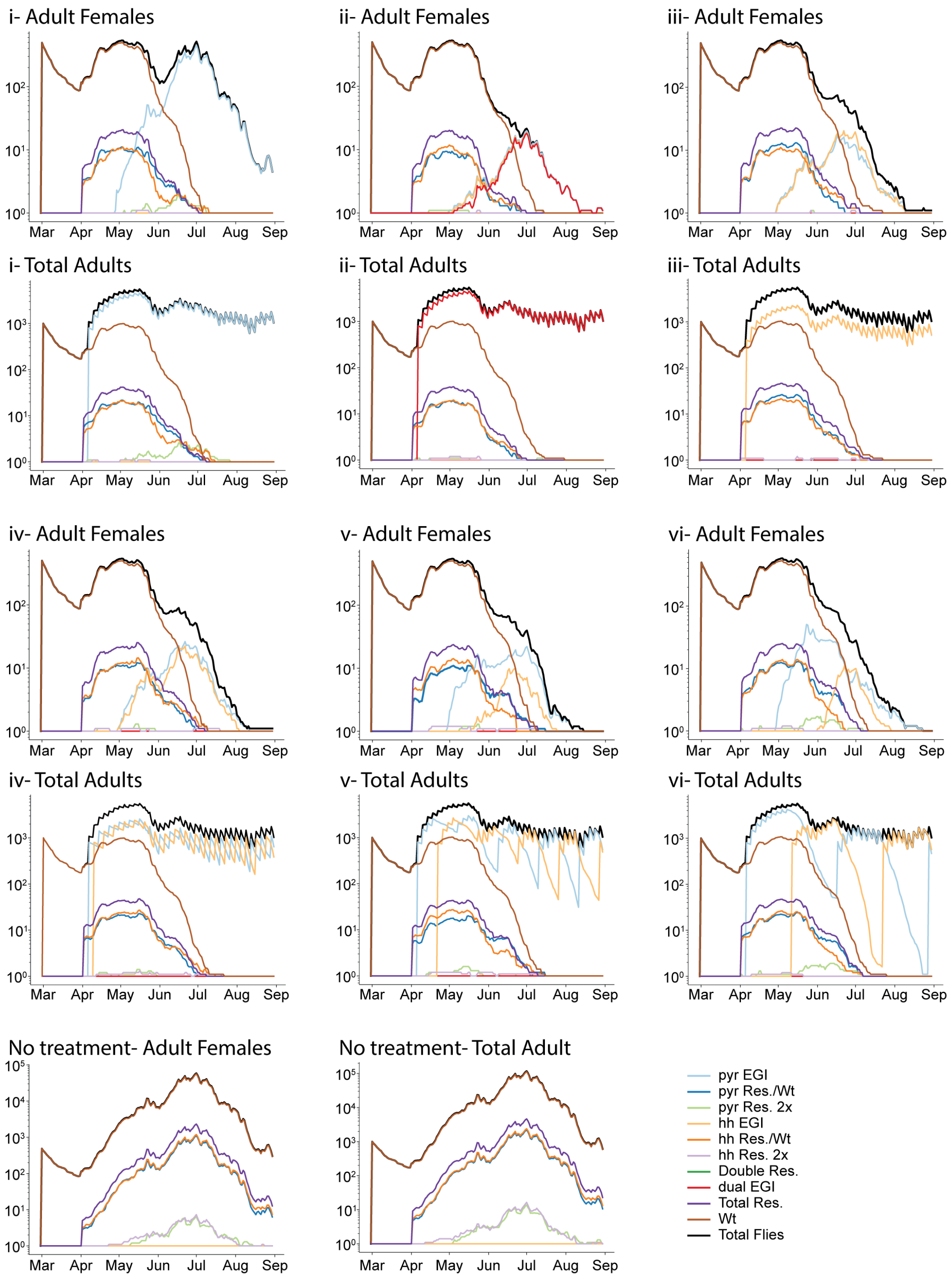

Fig. 6

Supplementary Note 3: Calculated parameters of logistic fit for ratio of EGI/Total females

Supp Note 3. a) Data represented in Figure 2d, EGI/Total females with 1% resistant allele frequency, fit to logistic equation. b)Data represented in Figure 2f, EGI/Total females with 1% double homozygous resistant allele frequency, fit to logistic equation.

a. Release Strategy	Midpoint (days)	Steepness (days^{-1})	R^2	RMSE
i:	101.60864926359258	0.10065336661731526	0.9931454334132684	0.03592179194345035
ii:	111.85472588133217	0.2094087930770341	0.9995127350803946	0.009087792402988418
iii:	114.18178708417155	0.09109822759908082	0.9945987434870305	0.02655590859393616
iv:	112.23404241905867	0.09040917041544592	0.9925922643990072	0.03180840092659953
v:	115.58108056702838	0.08310250004015168	0.9776908602455955	0.050307388649576706
vi:	114.18178708417155	0.09109822759908082	0.9945987434870305	0.02655590859393616

b. Release Strategy	Midpoint (days)	Steepness (days^{-1})	R^2	RMSE
i:	99.9961084575526	0.08157233564425	0.987246200113561	0.04692256319381519
ii:	98.00663904486706	0.151055264724273	0.999506768630059	0.009664898543863185
iii:	110.208410885356	0.072578508851382	0.988209208341172	0.039242920870340606
iv:	110.352307159336	0.07332517086638	0.992290306681313	0.032267688148533834
v:	114.122706295404	0.0622140124515	0.983546233880807	0.04692256319381519
vi:	119.207081869102	0.050352490658801	0.984303701467269	0.03666053169912254

Supplementary Note 4: Traces of adult genotypes over one season of treatment with high initial double resistance

Supp Note 4. Average counts of genotypes over one season seeded with 1% homozygous for both resistant alleles and treated according to Figure 2a.

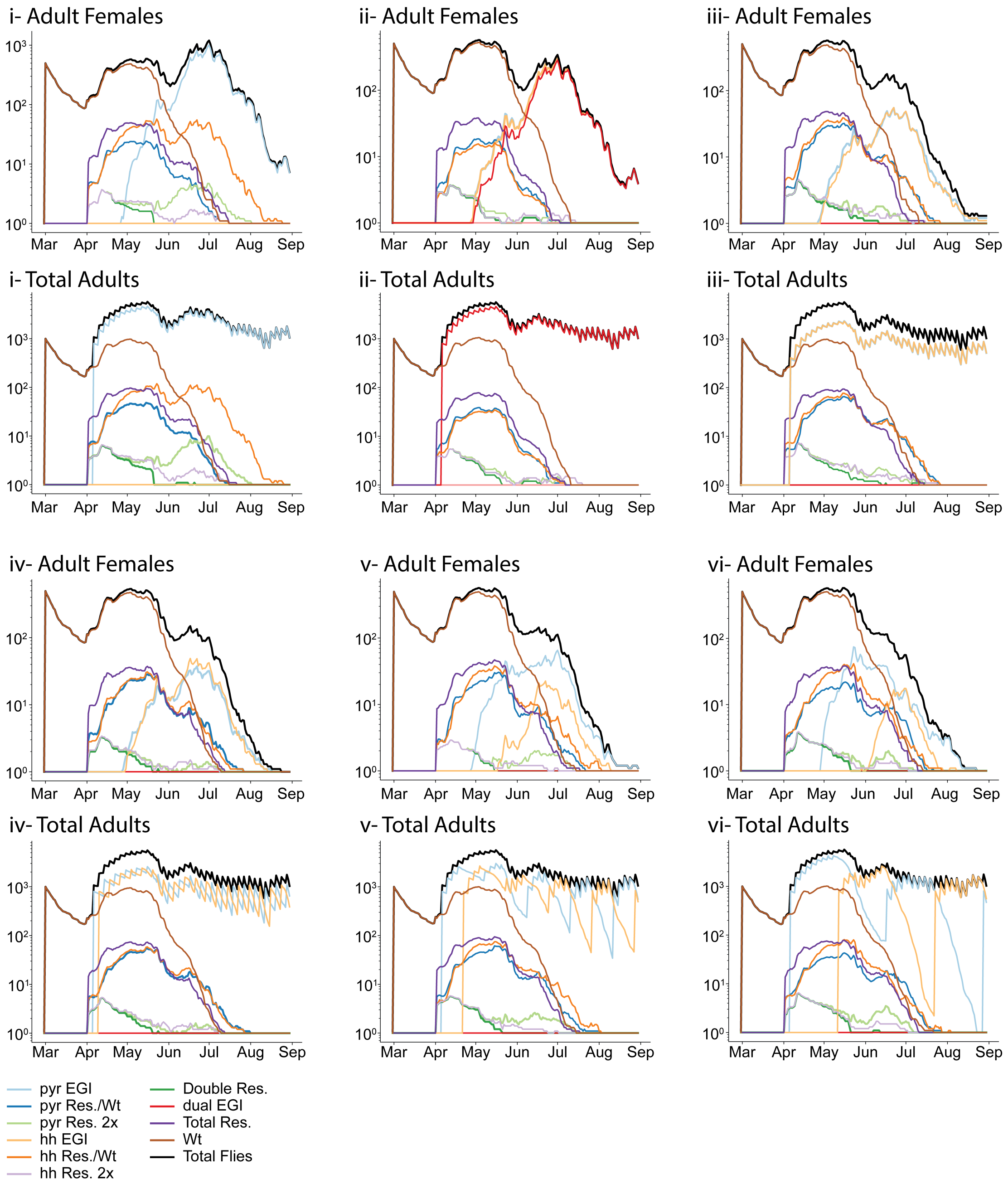

Fig. 7

Supplementary Note 5: Traces of adult genotypes over a second season of treatment

Supp Note 5. Average counts of genotypes over a seeded with with 1% resistant allele frequency and allowing overwintering. To model overwintering, 5, 50, or 95% of the initially seeded population was either the pyr EGI or dual EGI. The second season was treated according to Figure 2a with additional treatment where timing of pyr EGI and hh EGI were reversed.

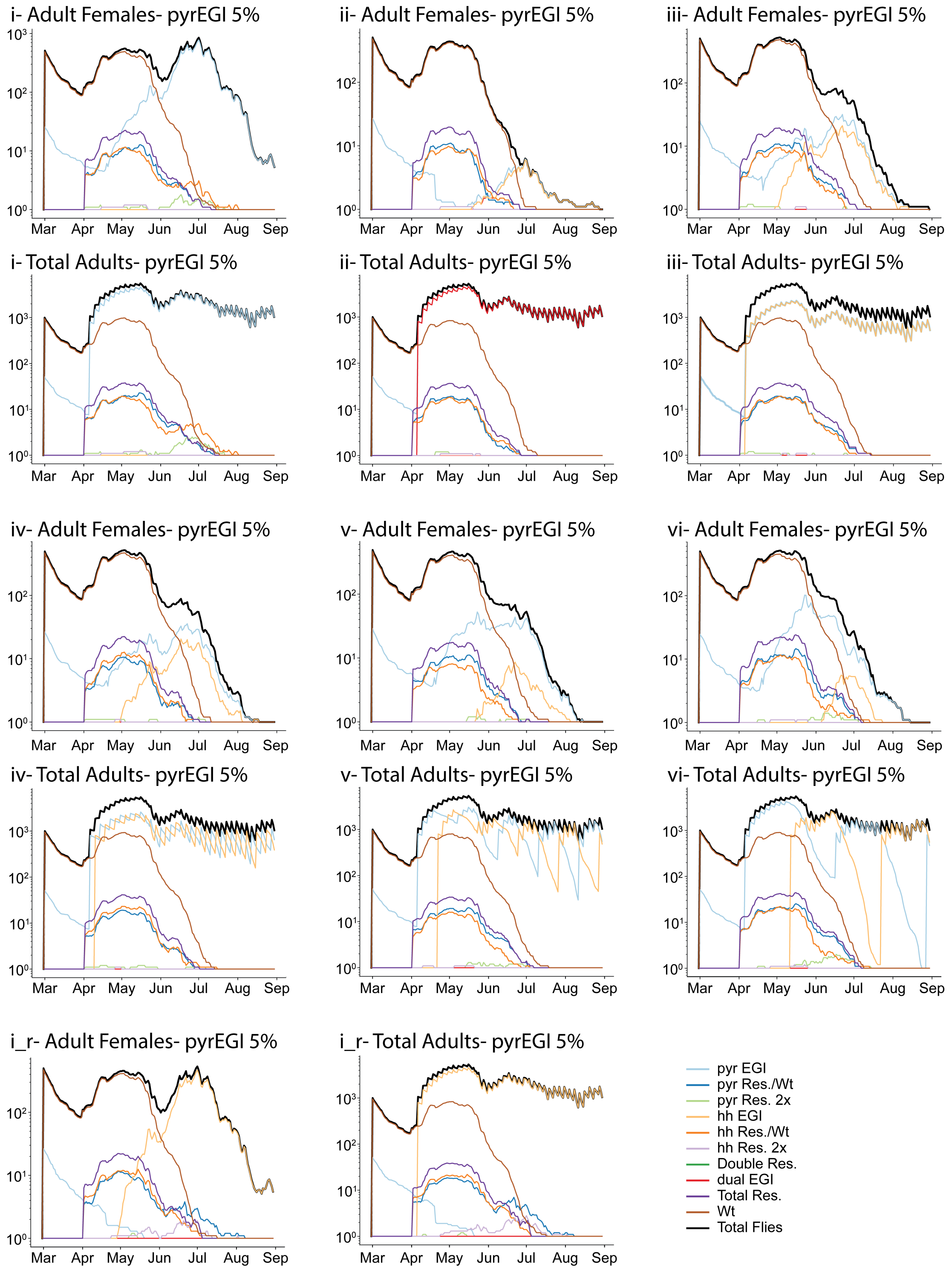

Fig. 8

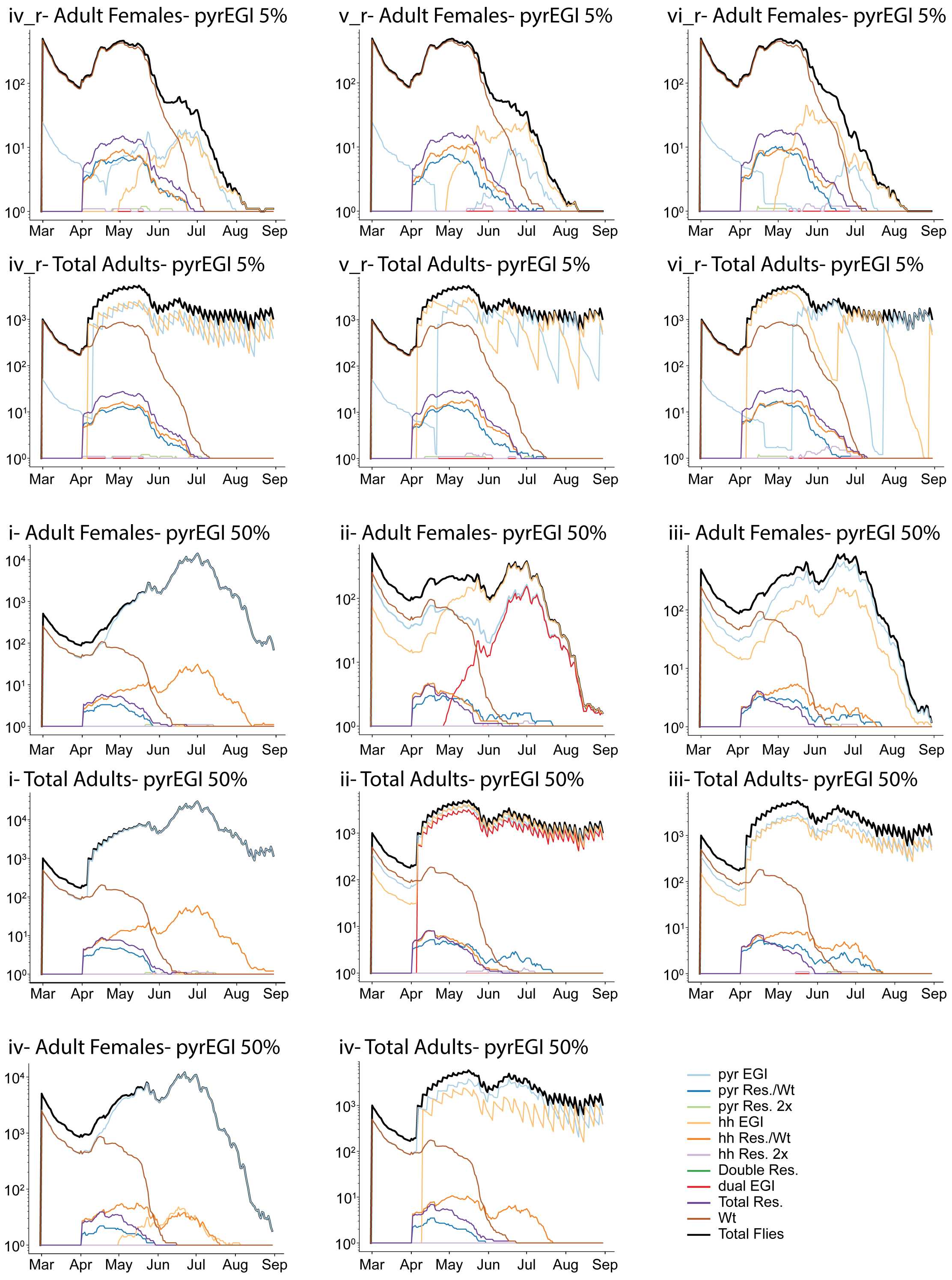
iv_r- Adult Females- pyrEGI 5%
v_r- Adult Females- pyrEGI 5%
vi_r- Adult Females- pyrEGI 5%
iv_r- Total Adults- pyrEGI 5%
v_r- Total Adults- pyrEGI 5%
vi_r- Total Adults- pyrEGI 5%
i- Adult Females- pyrEGI 50%
ii- Adult Females- pyrEGI 50%
iii- Adult Females- pyrEGI 50%
i- Total Adults- pyrEGI 50%
ii- Total Adults- pyrEGI 50%
iii- Total Adults- pyrEGI 50%
iv- Adult Females- pyrEGI 50%
iv- Total Adults- pyrEGI 50%
Mar Apr May Jun Jul Aug Sep
pyr EGI
pyr Res./Wt
pyr Res. 2x
hh EGI
hh Res./Wt
hh Res. 2x
Double Res.
dual EGI
Total Res.
Wt
Total Flies

Fig. 9

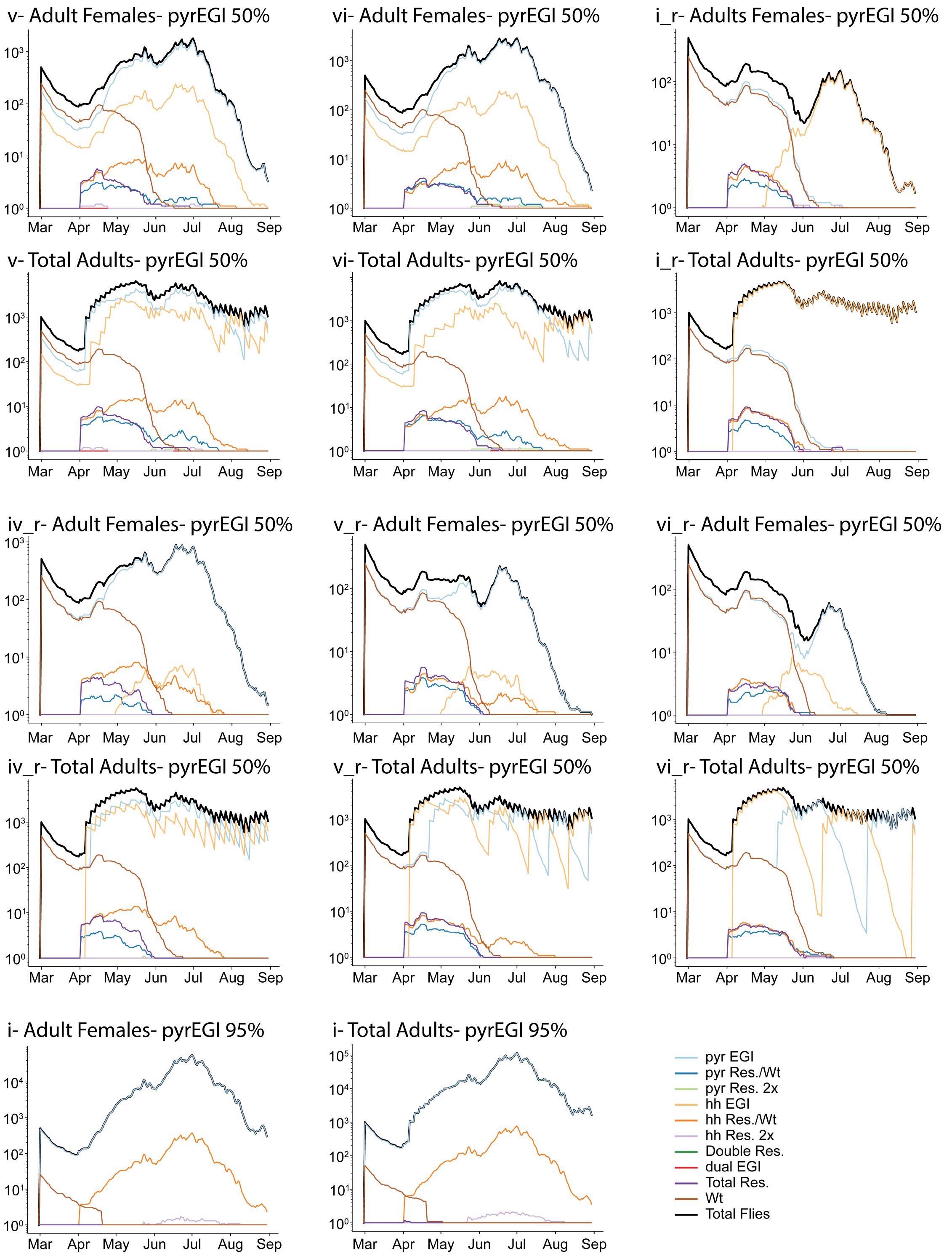
v- Adult Females- pyrEGI 50%
vi- Adult Females- pyrEGI 50%
i_r- Adults Females- pyrEGI 50%
v- Total Adults- pyrEGI 50%
vi- Total Adults- pyrEGI 50%
i_r- Total Adults- pyrEGI 50%
iv_r- Adult Females- pyrEGI 50%
v_r- Adult Females- pyrEGI 50%
vi_r- Adult Females- pyrEGI 50%
iv_r- Total Adults- pyrEGI 50%
v_r- Total Adults- pyrEGI 50%
vi_r- Total Adults- pyrEGI 50%
i- Adult Females- pyrEGI 95%
i- Total Adults- pyrEGI 95%
Mar Apr May Jun Jul Aug Sep
pyr EGI
pyr Res./Wt
pyr Res. 2x
hh EGI
hh Res./Wt
hh Res. 2x
Double Res.
dual EGI
Total Res.
Wt
Total Flies

Fig. 10

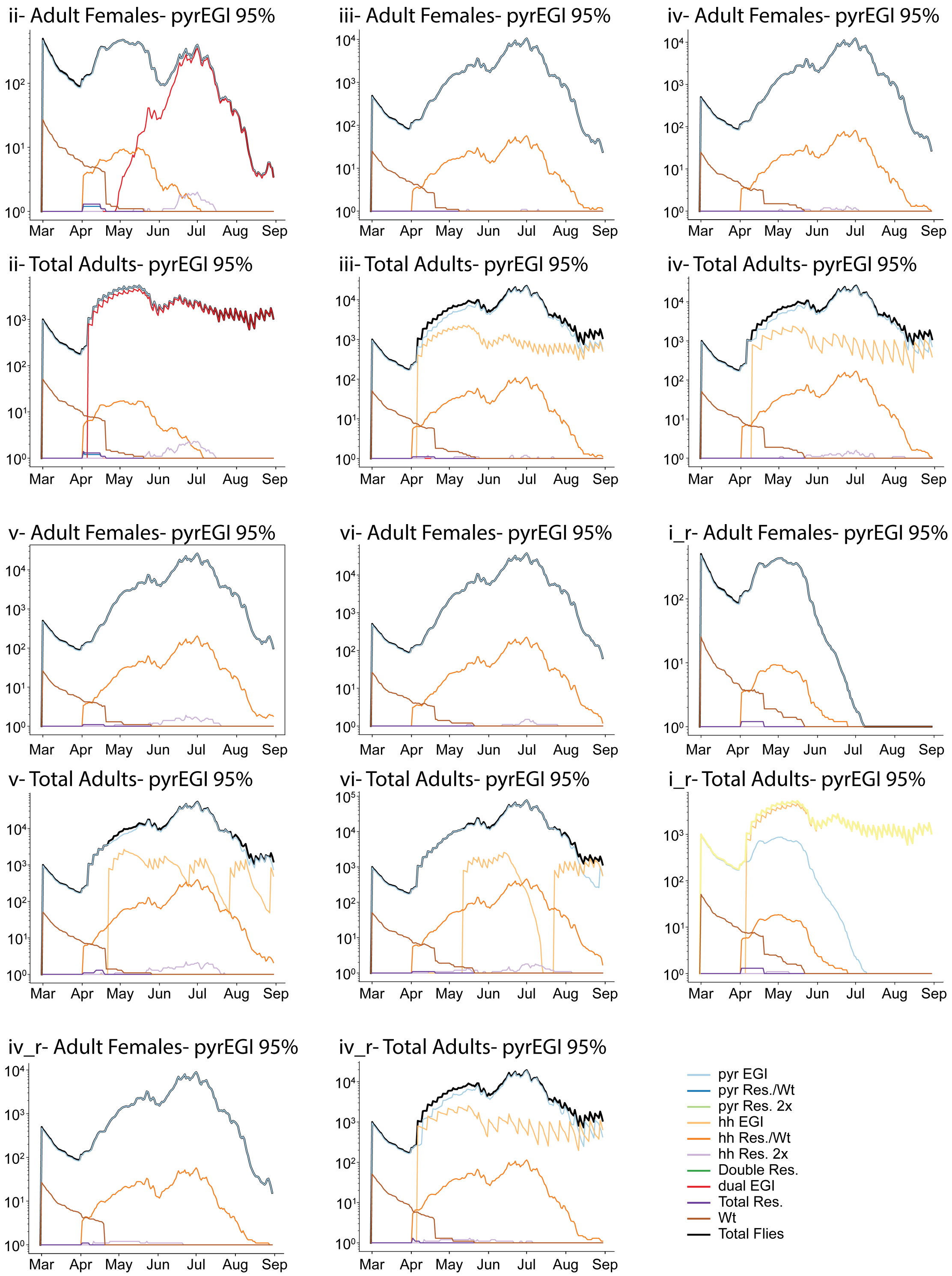
ii- Adult Females- pyrEGI 95%
iii- Adult Females- pyrEGI 95%
iv- Adult Females- pyrEGI 95%
ii- Total Adults- pyrEGI 95%
iii- Total Adults- pyrEGI 95%
iv- Total Adults- pyrEGI 95%
v- Adult Females- pyrEGI 95%
vi- Adult Females- pyrEGI 95%
i_r- Adult Females- pyrEGI 95%
v- Total Adults- pyrEGI 95%
vi- Total Adults- pyrEGI 95%
i_r- Total Adults- pyrEGI 95%
iv_r- Adult Females- pyrEGI 95%
iv_r- Total Adults- pyrEGI 95%
Mar Apr May Jun Jul Aug Sep
pyr EGI
pyr Res./Wt
pyr Res. 2x
hh EGI
hh Res./Wt
hh Res. 2x
Double Res.
dual EGI
Total Res.
Wt
Total Flies

Fig. 11

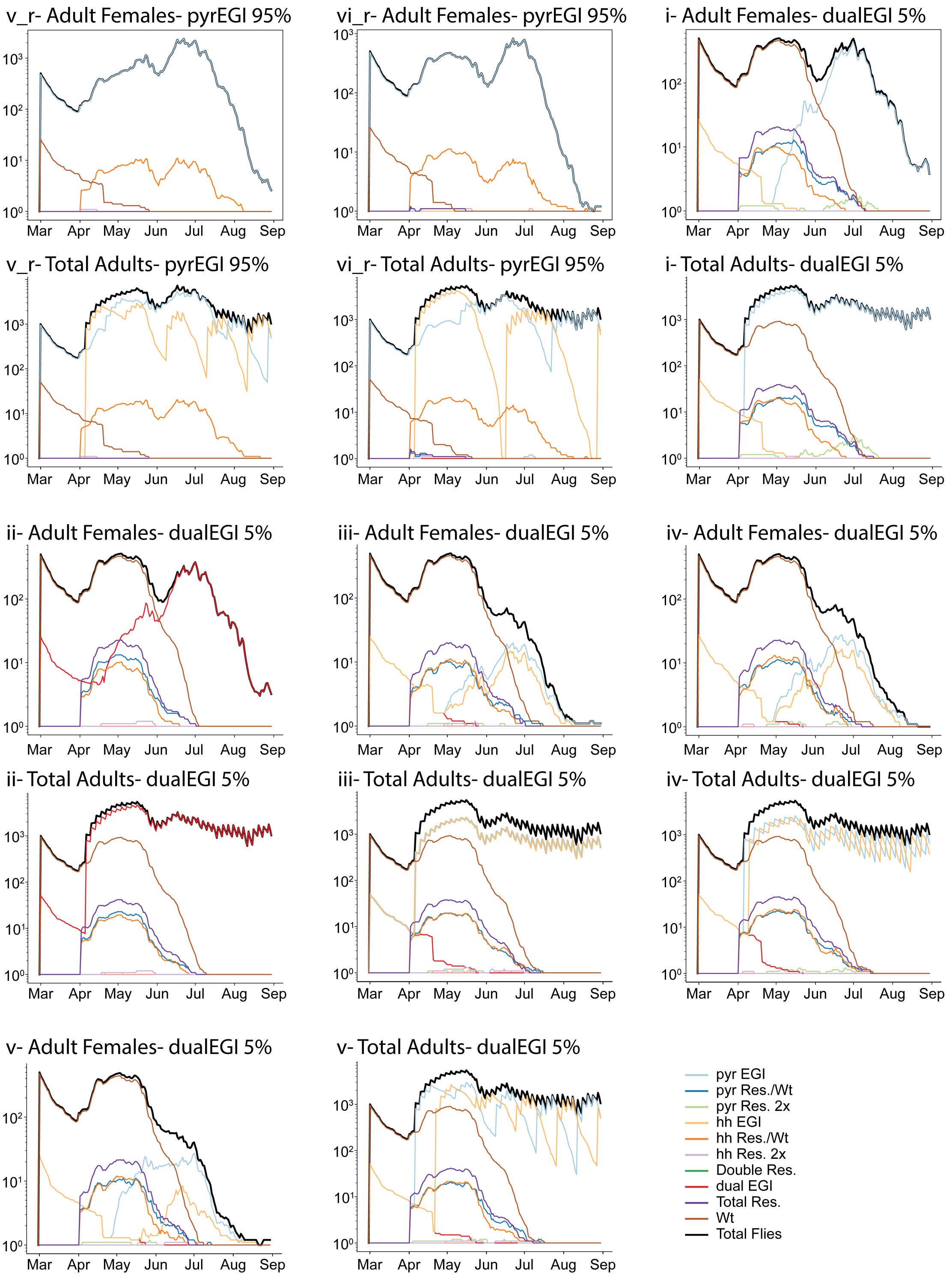

v_r- Adult Females- pyrEGI 95%
vi_r- Adult Females- pyrEGI 95%
i- Adult Females- dualEGI 5%
v_r- Total Adults- pyrEGI 95%
vi_r- Total Adults- pyrEGI 95%
i- Total Adults- dualEGI 5%
ii- Adult Females- dualEGI 5%
iii- Adult Females- dualEGI 5%
iv- Adult Females- dualEGI 5%
ii- Total Adults- dualEGI 5%
iii- Total Adults- dualEGI 5%
iv- Total Adults- dualEGI 5%
v- Adult Females- dualEGI 5%
v- Total Adults- dualEGI 5%
Mar Apr May Jun Jul Aug Sep
pyr EGI
pyr Res./Wt
pyr Res. 2x
hh EGI
hh Res./Wt
hh Res. 2x
Double Res.
dual EGI
Total Res.
Wt
Total Flies

Fig. 12

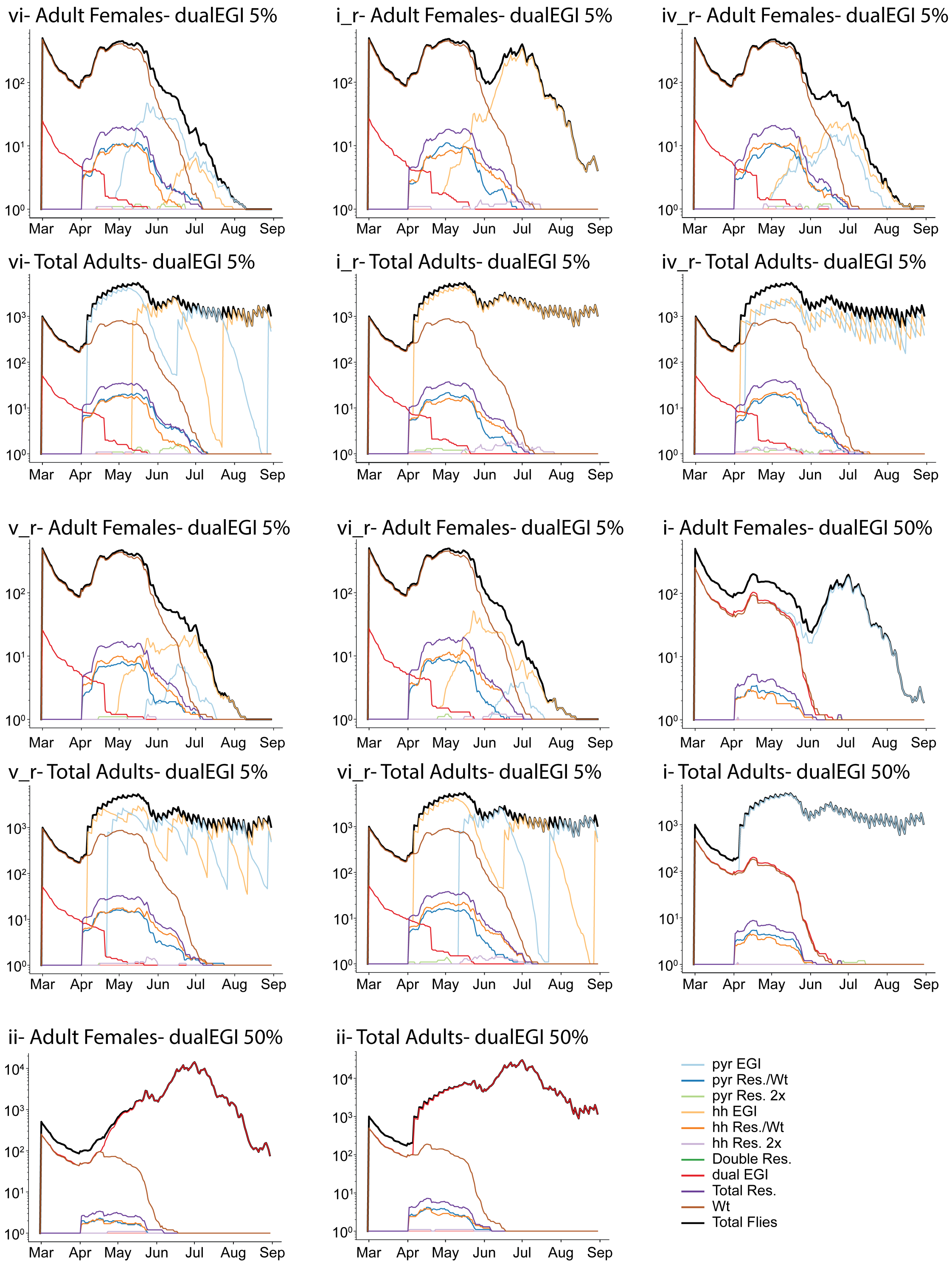
vi- Adult Females- dualEGI 5%
i_r- Adult Females- dualEGI 5%
iv_r- Adult Females- dualEGI 5%
vi- Total Adults- dualEGI 5%
i_r- Total Adults- dualEGI 5%
iv_r- Total Adults- dualEGI 5%
v_r- Adult Females- dualEGI 5%
vi_r- Adult Females- dualEGI 5%
i- Adult Females- dualEGI 50%
v_r- Total Adults- dualEGI 5%
vi_r- Total Adults- dualEGI 5%
i- Total Adults- dualEGI 50%
ii- Adult Females- dualEGI 50%
ii- Total Adults- dualEGI 50%
Mar Apr May Jun Jul Aug Sep
pyr EGI
pyr Res./Wt
pyr Res. 2x
hh EGI
hh Res./Wt
hh Res. 2x
Double Res.
dual EGI
Total Res.
Wt
Total Flies

Fig. 13

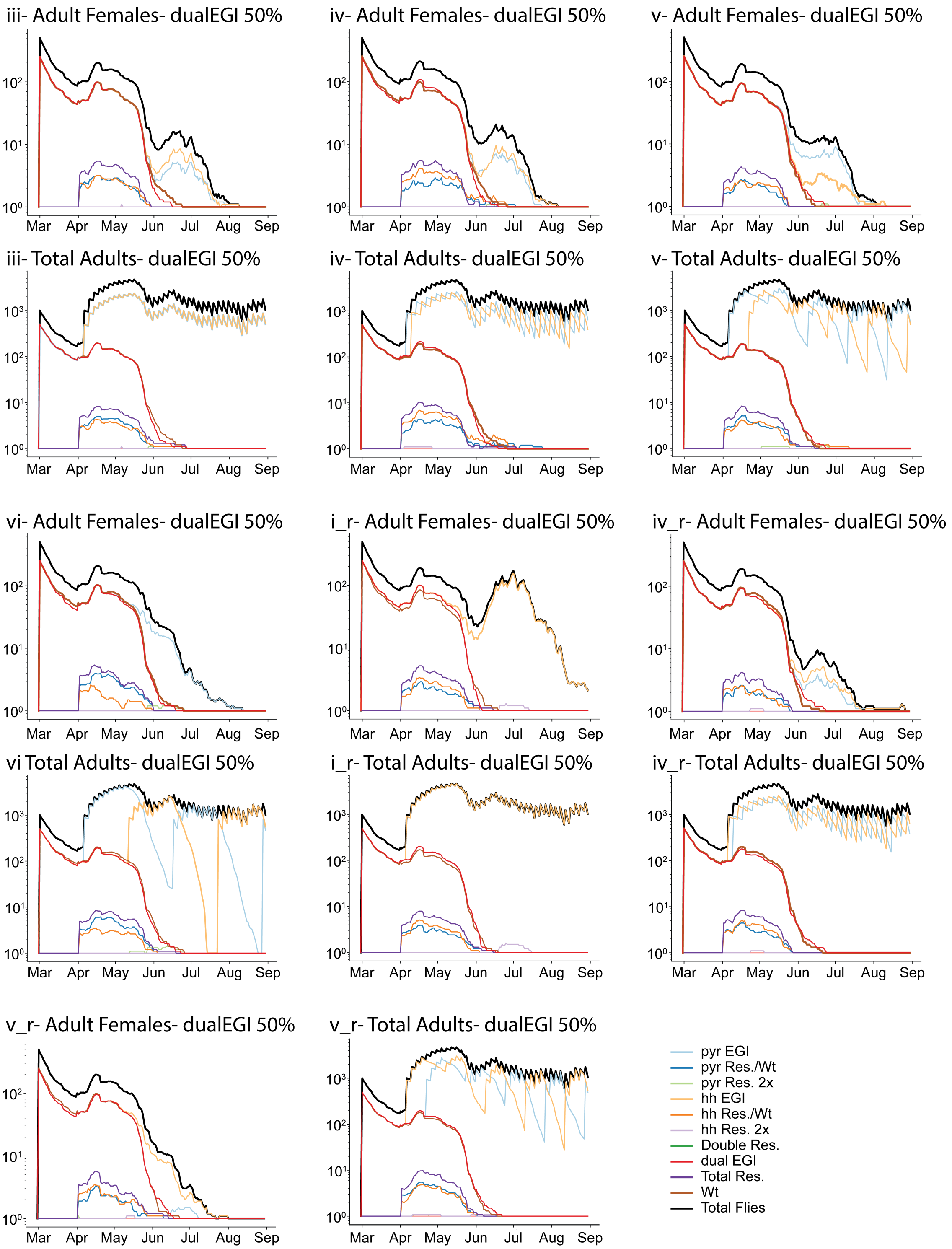

iii- Adult Females- dualEGI 50%
iv- Adult Females- dualEGI 50%
v- Adult Females- dualEGI 50%
iii- Total Adults- dualEGI 50%
iv- Total Adults- dualEGI 50%
v- Total Adults- dualEGI 50%
vi- Adult Females- dualEGI 50%
i_r- Adult Females- dualEGI 50%
iv_r- Adult Females- dualEGI 50%
vi Total Adults- dualEGI 50%
i_r- Total Adults- dualEGI 50%
iv_r- Total Adults- dualEGI 50%
v_r- Adult Females- dualEGI 50%
v_r- Total Adults- dualEGI 50%
Mar Apr May Jun Jul Aug Sep
pyr EGI
pyr Res./Wt
pyr Res. 2x
hh EGI
hh Res./Wt
hh Res. 2x
Double Res.
dual EGI
Total Res.
Wt
Total Flies

Fig. 14

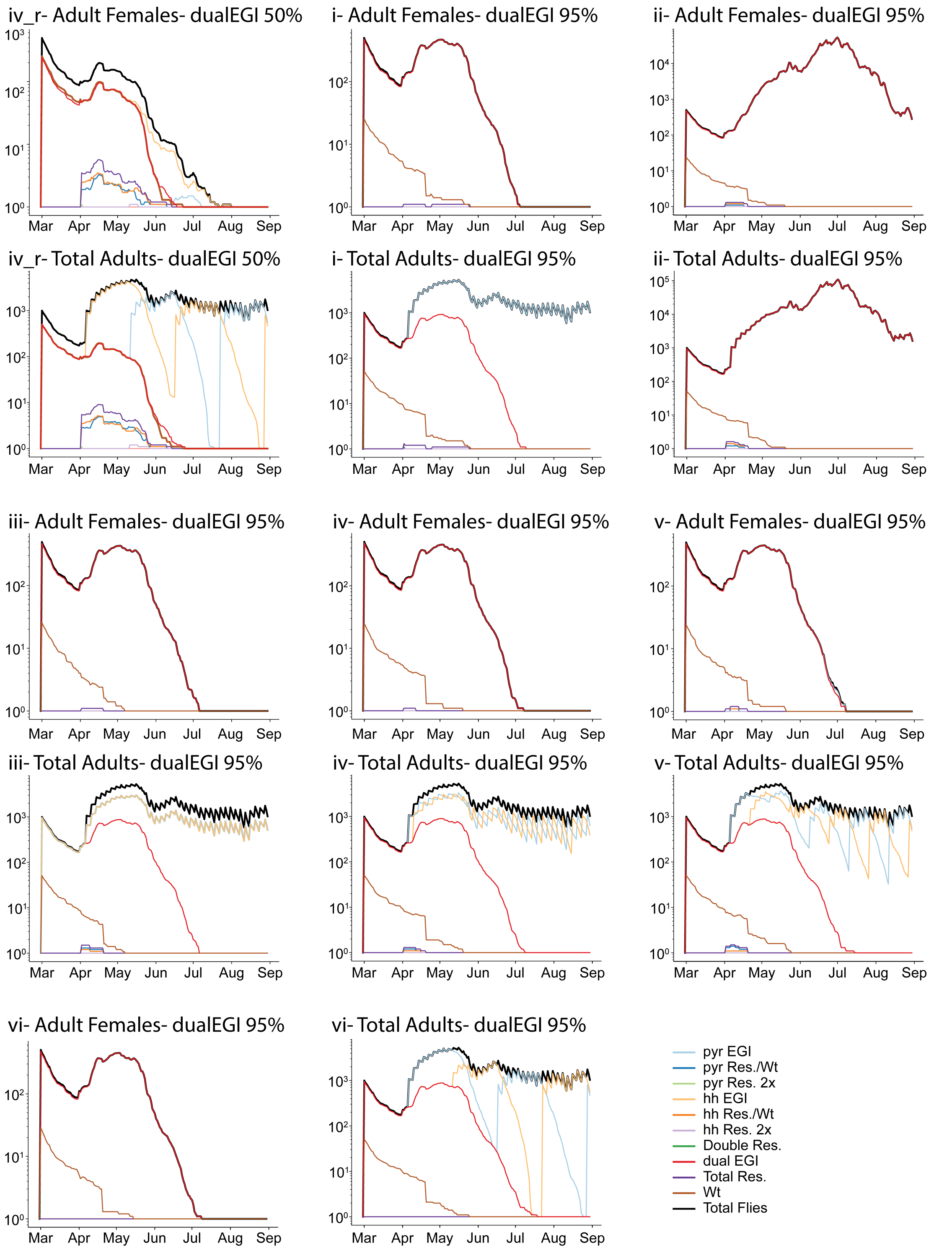
iv_r- Adult Females- dualEGI 50%
i- Adult Females- dualEGI 95%
ii- Adult Females- dualEGI 95%
iv_r- Total Adults- dualEGI 50%
i- Total Adults- dualEGI 95%
ii- Total Adults- dualEGI 95%
iii- Adult Females- dualEGI 95%
iv- Adult Females- dualEGI 95%
v- Adult Females- dualEGI 95%
iii- Total Adults- dualEGI 95%
iv- Total Adults- dualEGI 95%
v- Total Adults- dualEGI 95%
vi- Adult Females- dualEGI 95%
vi- Total Adults- dualEGI 95%
pyr EGI
pyr Res./Wt
pyr Res. 2x
hh EGI
hh Res./Wt
hh Res. 2x
Double Res.
dual EGI
Total Res.
Wt
Total Flies

Fig. 15

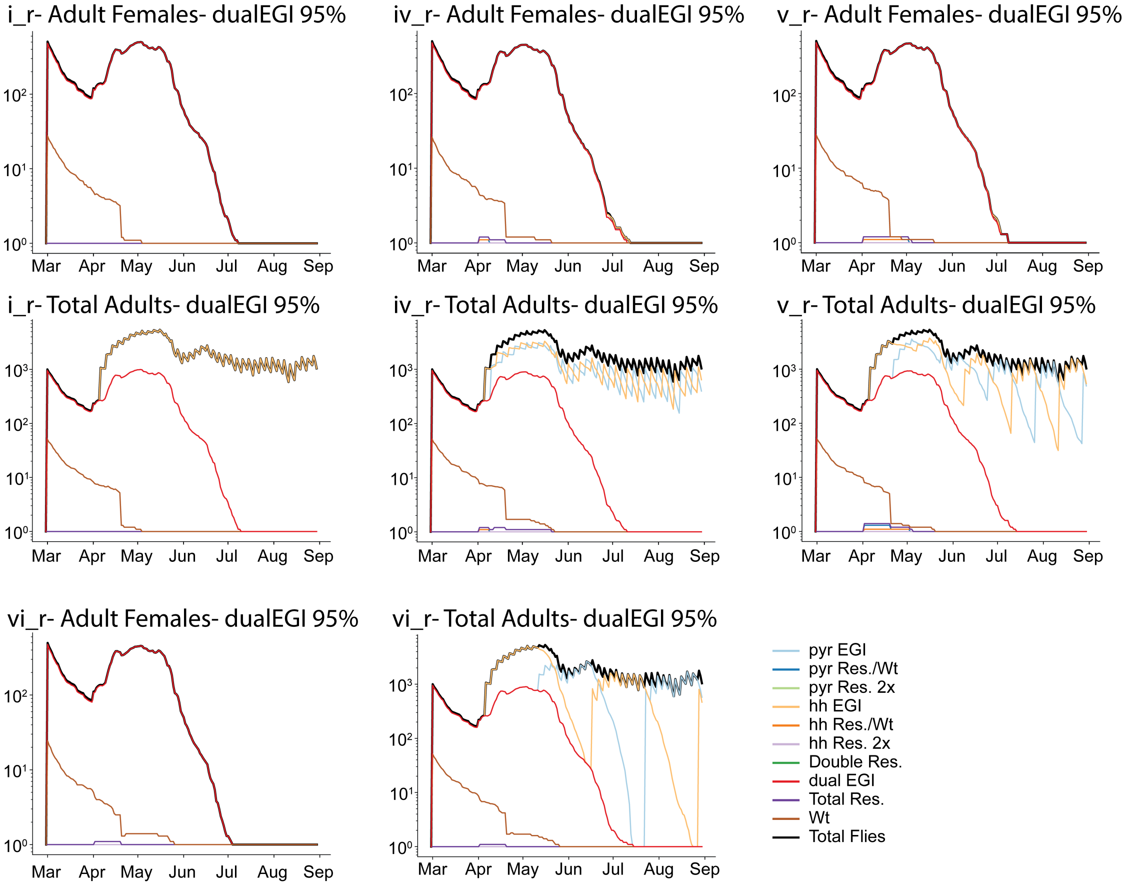
i_r- Adult Females- dualEGI 95%
iv_r- Adult Females- dualEGI 95%
v_r- Adult Females- dualEGI 95%
i_r- Total Adults- dualEGI 95%
iv_r- Total Adults- dualEGI 95%
v_r- Total Adults- dualEGI 95%
vi_r- Adult Females- dualEGI 95%
vi_r- Total Adults- dualEGI 95%
Mar Apr May Jun Jul Aug Sep
pyr EGI
pyr Res./Wt
pyr Res. 2x
hh EGI
hh Res./Wt
hh Res. 2x
Double Res.
dual EGI
Total Res.
Wt
Total Flies

Fig. 16

Supplementary Note 6: Traces of adult genotypes over an L-SSIMS treatment with no FL resistance

Supp Note 6. Average counts of genotypes over one season seeded with 1% EGI resistant allele frequency and treated with pyr L-SSIMS.

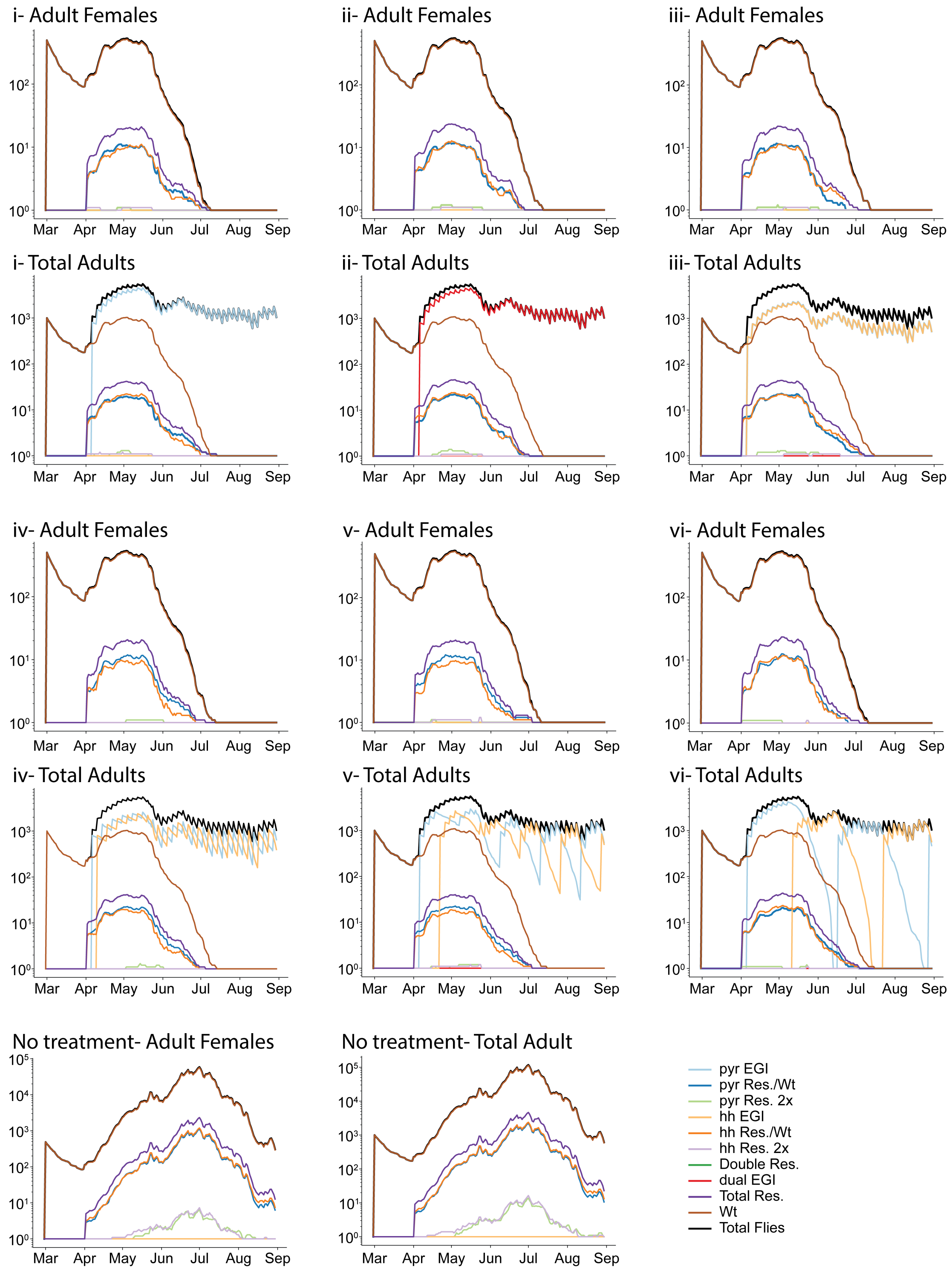

Fig. 17

Supplementary Note 7: Traces of adult genotypes over an L-SSIMS treatment with 10% PTA and 10% FL reversion

Supp Note 7. Average counts of genotypes over one season seeded with 1% resistant allele frequency and treated with pyr L-SSIMS. FL reverted to wild-type in 10% of inherited FL alleles. PTA reverted to wild-type in 10% of inherited PTA alleles.

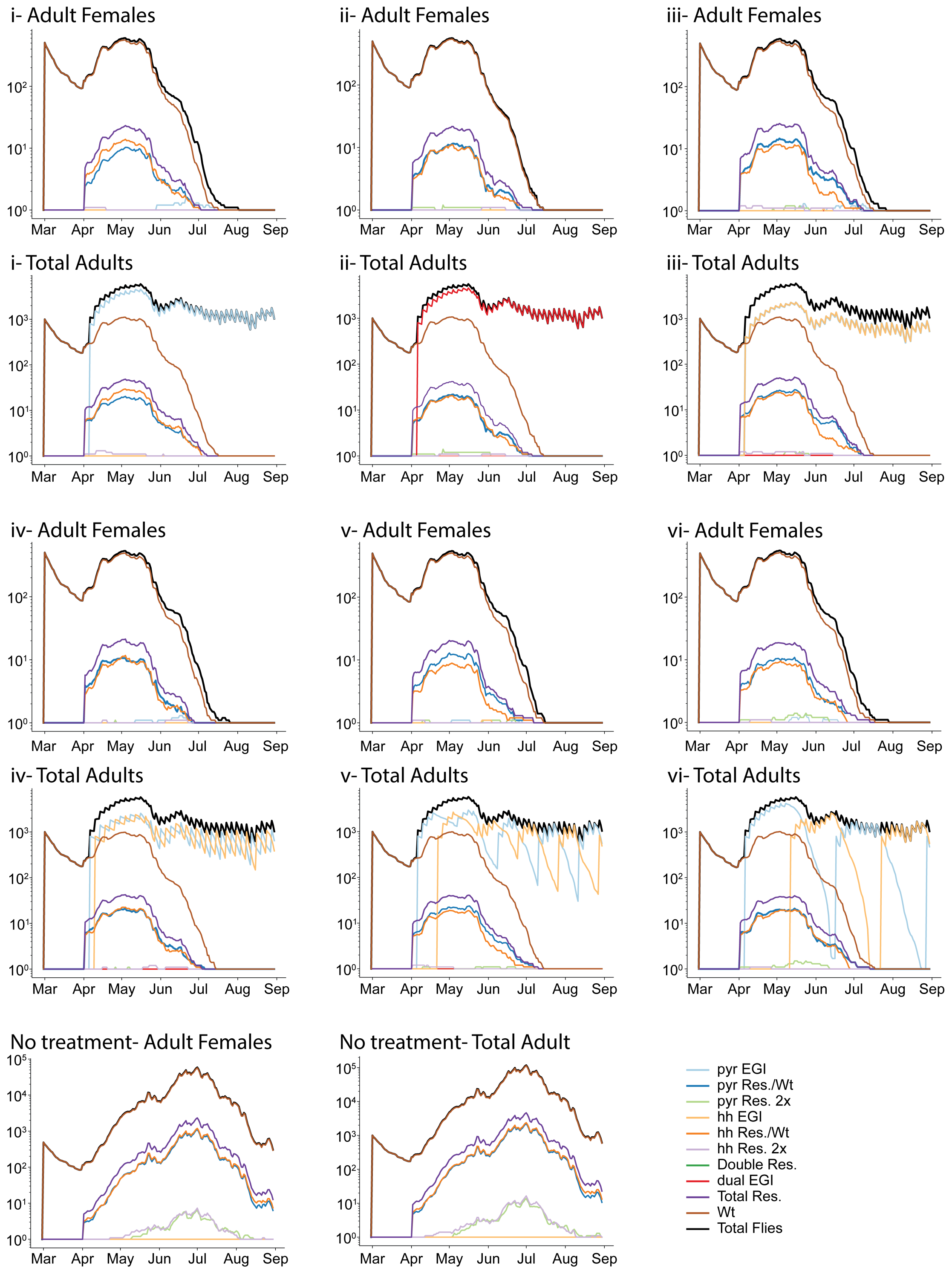

Fig. 18
